# Behavioral Impairment and Oxidative Damage Induced by Chronic Application of Nonylphenol

**DOI:** 10.3390/ijms12010114

**Published:** 2010-12-30

**Authors:** Zhen Mao, Yuan-Lin Zheng, Yan-Qiu Zhang

**Affiliations:** 1 School of Environment Science and Spatial Informatics, China University of Mining and Technology, Xuzhou 221008, Jiangsu, China; E-Mail: yqzhang@cumt.edu.cn (Y.-Q.Z.); 2 Key Laboratory for Biotechnology on Medicinal Plants of Jiangsu Province, School of Life Science, Xuzhou Normal University, Xuzhou 221116, Jiangsu, China; E-Mail: ylzheng@xznu.edu.cn (Y.-L.Z.)

**Keywords:** nonylphenol, brain, oxidative stress, behavior impairment

## Abstract

Nonylphenol (NP) is a degradation product of nonylphenol polyethoxylates, which are widely used in the production of industrial and consumer surfactants. The aim of the present study was to evaluate the effect of NP on the antioxidant capacity and cognitive ability of mice. NP was given orally by gavages at doses of 0, 50, 100, and 200 mg kg^−1^ d^−1^ for 90 days. The results showed that NP significantly decreased the activity of superoxide dismutases (SOD), catalase (CAT), glutathione peroxidase (GPx), and glutathione reductase (GR) and at the same time increased malondialdehyde (MDA) levels in mice brains. Exploration, memory function and ability to learn a novel task were significantly decreased in NP fed mice. These results indicate that chronic high dose of NP exposure has the potential to generate oxidative stress and induce the cognitive impairment in male mice.

## 1. Introduction

During recent decades, reports of the identification of endocrine disruptors in the environment initiated concern over the use of such substances. Nonylphenol (NP) is one of the endocrine disruptors, and many *in vivo* studies have shown that NP can exert a generally chronic toxicity to the reproductive system [1–3], immune system [4,5], and the digestive system [6]. Our previous work showed that NP can induced apoptosis and chronic inflammation in mouse brain [7,8]. However, up until now, only little behavior data of the neurotoxicity of NP have been reported. In Flynn’s study [9], two generations of Sprague-Dawley rats were exposed to NP in the diet at 0, 25, 200 and 750 ppm, which corresponded to dietary intakes of 0, 2, 16, and 60 mg kg^−1^ d^−1^. No significant alterations were found in Morris water maze performance in young adult or middle aged ovariectomized female rats. In Negishi’s study [10], 0.1 mg kg^−1^ d^−1^ and 10 mg kg^−1^ d^−1^ NP were given to F344 rats from gestational day 3 to postnatal day 20 (transplacental and lactational exposures). NP exposure did not influence the behavioral characteristics in the open-field test, in a spontaneous motor activity test, or in an elevated plus-maze test in male offspring. However, NP exposure irreversibly influenced the reception of intolerable stress and responses to the disruption of the monoaminergic system. In our study, higher doses of NP (50–200 mg kg^−1^ d^−1^) were given to examine whether chronic exposure to NP would lead to behavioral alterations in male mice. The lower dose of 50 mg kg^−1^ d^−1^ in this study is close to the NOAEL (No Observed Adverse Effect Level; 50 mg kg^−1^ d^−1^) of rats [11] but lower than that of mice. The highest dose of 200 mg kg^−1^ d^−1^ approaches the maximum tolerated dose that could be used without visible signs of toxicity.

The brain is particularly vulnerable to oxidative stress. Antioxidant enzymes including superoxide dismutase (SOD), catalase (CAT), glutathione peroxidase (GPx) and glutathione reductase (GR) protect the brain from oxidative damage [12]. SODs catalyze the dismutation of superoxide into oxygen and hydrogen peroxide, thus protecting the cell from superoxide toxicity. CAT is the most common enzyme in nearly all living organisms exposed to oxygen. It catalyzes the decomposition of hydrogen peroxide to water and oxygen. GPx catalyses the reduction of hydroxyl peroxides by glutathione, and protects against the damage of hydroxyl peroxides. GR is an enzyme that reduces glutathione disulfide (GSSG) to the sulfhydryl form glutathione, which is used as an indicator for oxidative stress.

Oxidative damage plays an important role in the pathogenesis of various CNS disorders [13] and neurobehavioral impairments [14]. In recent years, accumulating evidence indicated that NP induces ROS generation in human neutrophils [15], rat Sertoli cells [16], *Escherichia coli* mutant cells [17] and rat liver [6]. Although Aydogan M. has showed that malondialdehyde (MDA) concentration was increased and glutathione concentration was decreased in the brain of rats exposed to NP [18], other brain oxidative stress-related parameters such as SOD, CAT and GPx were not evaluated in his study. In addition, it was not revealed whether the oxidative stress can induce other impairments. Therefore, the present study was designed to investigate the effects of chronic NP administration on oxidative stress and neurobehavior in mouse brain.

## 2. Results and Discussion

### 2.1. Effects of NP on the Behavior of Mice

#### 2.1.1. Open Field Test

**Figure 1:**
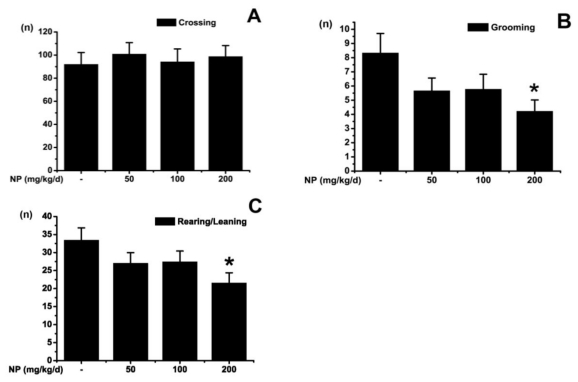
The locomotor and exploratory behavior of nonylphenol (NP) treated mice at different doses or vehicle (*n* = 9). All values are expressed as means ± S.E.M. (**A**) Comparison of crossing numbers (within 5 min); (**B**) Comparison of rearing/leaning numbers (within 5 min); (**C**) Comparison of grooming numbers (within 5 min). * *p* < 0.05 *vs.* the vehicle control.

The open field test is a common measure of exploratory behavior and general activity [19]. As illustrated in Figure 1, the numbers of grids crossed in the testing apparatus was not different among groups (*p* > 0.05). The activities of rearing/leaning (*p* < 0.05) and grooming (*p* < 0.05) were significantly decreased in the group treated with 200 mg kg^−1^ d^−1^ NP. However, the groups treated with NP at 50 and 100 mg kg^−1^ d^−1^ showed no significant difference in rearing/leaning (*p* > 0.05) and grooming activity (*p* > 0.05) as compared with the control group. The numbers of crossing and rearing are usually used as measures of locomotor activity, and also measures of exploration and anxiety as well. A low frequency of these behaviors indicates decreased locomotion and exploration and/or a higher level of anxiety. Grooming behavior is a displacement response and is expected to be displayed in a novel environment in an open field test. Our results indicated that a high dose of NP exposure decreases locomotion and exploration of mice.

#### 2.1.2. Step-Through Test

**Figure 2:**
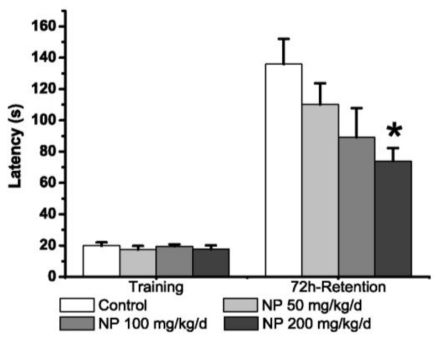
Performance in the step-through tasks (*n* = 9). All values are expressed as means ± S.E.M. * *p* < 0.05 *vs.* vehicle control.

In the acquisition trial, the latencies for entry into the dark compartment did not differ significantly among groups (*p* > 0.05) (Figure 2). In the 72-hour-retention trial, the latencies in mice treated with NP at 200 mg kg^−1^ d^−1^ were significantly decreased (*p* < 0.05), but the decrease was not significant in the groups at 50 and 100 mg kg^−1^ d^−1^ compared with the control group (*p* > 0.05).

#### 2.1.3. Morris Water Maze Test

**Figure 3:**
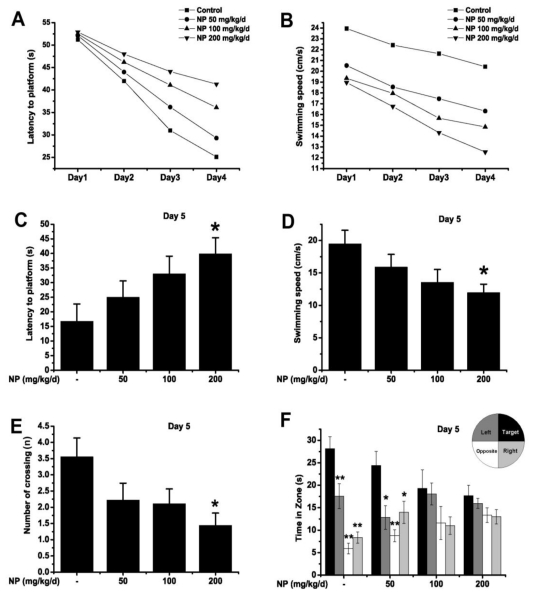
The Morris water maze test of NP treated mice at different doses or vehicle (*n* = 9). All values are expressed as means ± S.E.M. (**A**) Comparison of latencies to platform during 4 training days; (**B**) Comparison of swimming speeds during 4 training days. Each mouse was subjected to four trials per day; (**C**) Comparison of latencies to platform on Day 5; (**D**) Comparison of swimming speeds on Day 5; (**E**) Comparison of numbers of crossing over the platform site on Day 5. * *p* < 0.05 *vs.* vehicle control; (**F**) Comparison of the time spent in the target quadrant with other quadrants on Day 5. * *p* < 0.05; ** *p* < 0.01 *vs.* target quadrant.

The Morris water maze test was used to reveal the effects of NP on spatial learning and memory. The mean latency to find the platform declined progressively during the training days in all groups (Figure 3A). Significant differences were found in mean latencies between training days (*p* < 0.01) and between treatments (*p* < 0.01), but there was no interaction between the factors day and treatment (*p* > 0.05). The escape latencies were significantly higher in NP (200 mg kg^−1^ d^−1^) treated mice than in the control mice (Day 3: *p* < 0.05; Day 4: *p* < 0.01). As there was no significant difference between the groups on Day 1, mice of every group started at the same level of performance. The mean swimming speeds of differently treated mice during the training days are presented in Figure 3B. Mice also showed significant differences in swimming speeds between training days (*p* < 0.01) and between treatments (*p* < 0.01), while no interaction between the factors day and treatment (*p* > 0.05) were observed. Swimming speeds of NP (200 mg kg^−1^ d^−1^) treated mice were significantly decreased compared to the control group (Day 1–Day 4: *p* < 0.05). This indicates that the motor problem of mice may contribute to the latency differences during the training days. This shows that NP could impair the locomotor activity of mice, leading to extended latency to the platform.

On Day 5, latency, swimming speed, the number of times crossing over the platform site and the time spent in four quadrants were measured for all groups. The NP-treated mice (at the dose of 200 mg kg^−1^ d^−1^) had longer latencies (*p* < 0.05) and a slower swimming speed (*p* < 0.05) compared with the control (Figures 3C and 3D). In the probe trial, the NP (200 mg kg^−1^ d^−1^) treated group made fewer platform crossings than the control group (*p* < 0.05) (Figure 3E). Both 50 mg kg^−1^ d^−1^ NP-treated and control mice spent most of their time in the target quadrant where the platform had been located during the training (Figure 3F).On the other hand, the groups treated with 100 and 200 mg kg^−1^ d^−1^ NP showed a decreased spatial preference for the target quadrant as compared with the control. All results revealed that NP at a higher dose (200 mg kg^−1^ d^−1^) could impair the ability of spatial learning and memory as well as the locomotor activity of mice.

### 2.2. Effects of NP on Antioxidative Status of Mouse Brain

**Figure 4:**
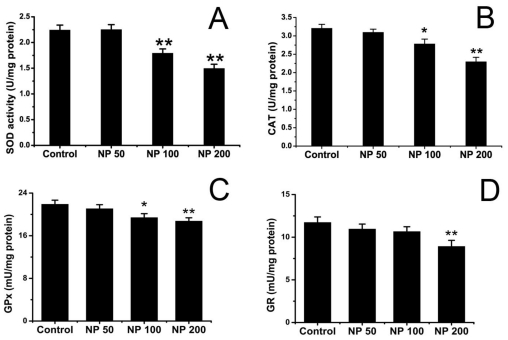
The effect of NP on SOD (**A**), CAT (**B**), GPx (**C**) and GR (**D**) activity in mouse brain (*n* = 9). Values are expressed as mean ± S.E.M. * *p* < 0.05; ** *p* < 0.01 *vs.* the vehicle control.

Figure 4 shows the effects of NP on SOD, CAT, GPx and GR activity of mouse brain. The activities of SOD, catalase and GPx in the brain decreased significantly in mice treated with NP at doses of 100 and 200 mg kg^−1^ d^−1^ as compared with the control group (*p* < 0.05). No significant difference was detected in the group treated with 50 mg kg^−1^ d^−1^ NP in terms of SOD, CAT and GPx activity (*p* > 0.05). NP at the dose of 200 mg kg^−1^ d^−1^ decreased the activity of GR in the brain (*p* < 0.05) while 50 and 100 mg kg^−1^ d^−1^ NP did not reach this effect (*p* > 0.05).

### 2.3. Effects of NP on Lipid Peroxidation Level of Mouse Brain

**Figure 5:**
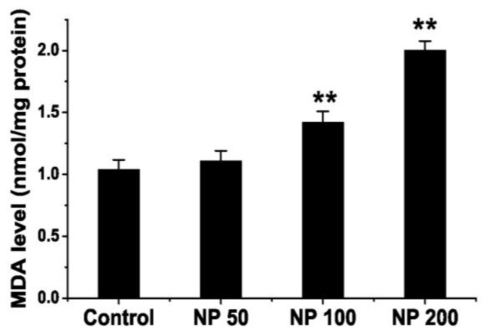
The effect of NP on malondialdehyde (MDA) level in mouse brain (*n* = 9). Values are expressed as mean ± S.E.M. ** *p* < 0.01 *vs.* the vehicle control.

As a marker of lipid peroxidation, the level of MDA was examined. The MDA level increased in the groups treated with NP at doses of 100 and 200 mg kg^−1^ d^−1^ (*p* < 0.01), but the increase was not significant at a dose of 50 mg kg^−1^ d^−1^ as compared with the control group (*p* > 0.05) (Figure 5).

### 2.4. Discussion

Since hormonally mediated events play a central role in central nervous system development and function, endocrine disrupters were speculated to make changes in cognitive function by the endocrine-like actions [20]. Some of them have been shown to influence the cognitive function in animals, such as polychlorinated biphenyls (PCBs) [21,22]. In a four-day metabolic balance study [23], Wistar rats were given orally 1 μg/kg (“low-dose”) or 10 mg/kg (“high-dose”) labeled 4-*n*-nonylphenol. Although most of the administered radioactivity was excreted in urine and feces, radioactivity was detected in the blood, liver, brain and other tissues. Radioactivity for samples from the high-dose group (dosed 10 mg/kg) was about 10,000-fold higher than recorded for the low-dose group (dosed 1 μg/kg). These results indicated that NP could reach the brain and may accumulate in it for a short time. The present study was designed to investigate the effects of chronic NP administration on neurobehavioral effect and the possible causes. A number of studies have been carried out examining the neurobehavioral effect of NP. These studies are difficult to compare directly, as they use a variety of designs and test systems. However, these previous studies indicated that low doses of NP (2–60 mg kg^−1^ d^−1^ or 0.1–10 mg kg^−1^ d^−1^) did not alter the behavioral characteristics in an open-field test [10] or Morris water maze test [9]. These results are in agreement with our studies that NP at a low dose (50 or even 100 mg kg^−1^ d^−1^) did not influence learning and memory in mice. However, a high dose of NP (200 mg kg^−1^ d^−1^) significantly decreased the exploration, memory function and ability of mice to learn a novel task. This biphasic effect of NP exposure might be due to the “inverted-U” effect, which is in accord with the dose-dependent biphasic (stimulatory then inhibitory) phenomenon, “hormesis”, that occurs below the NOAEL (no-observed-adverse-effect level) [24]. As for NP, the NOAEL is 50 mg kg^−1^ d^−1^ in rat; below or close to the NOAEL value, NP might induce a stimulatory effect whereas inhibitory phenomenon might emerge at high doses of NP.

Oxidative stress has been implicated as the prime candidate mediating behavioral impairment and memory deficits [25]. Due to high oxygen consumption and elevated metabolic activity, the brain is more vulnerable to ROS-induced neurotoxicity. In this study, we measured the activity of antioxidant defense enzymes (such as SOD, catalase, GPx and GR) to assess the oxidative stress status in the brain. Our results showed that the activities of SOD, CAT, GPx and GR were significantly decreased by NP. Only a very slight increase of SOD activity was observed in the brains of mice treated with NP at 50 mg kg^−1^ d^−1^ group (*p* > 0.05), which may be a compensatory increase after persistently increased oxidative stress. The glutathione system plays an important role in the defense system against ROS. GPx catalyzes the reduction of H_2_O_2_ by GSH into H_2_O and GSSG, and then GR recycles GSSG to GSH. During the reactions catalyzed by GPx and GR, glutathione is not consumed, but recycled. When cells are exposed to increased levels of oxidative stress, GSSG will accumulate and the ratio of GSH to GSSG will decrease. In our study, the GPx and GR activities were found to be significantly reduced after NP exposure, indicating that the cells are surmounted by ROS and antioxidant enzymes are inadequate to conquer ROS damage. Obata T. investigated effects of nonylphenol on hydroxyl radical formation in the striatum of adult rats, and found that nonylphenol induced hydroxy radical formation. The estrogenic action via estrogen receptors might be implicated in this effect [26].

The accumulation of ROS may induce lipid peroxidation. Brain is enriched in polyunsaturated fatty acids (PUFAs) that are susceptible to lipid peroxidation. As a biomarker of lipid peroxidation and oxidative stress, the MDA content was measured in all groups. NP significantly increased the MDA content at all the doses. These results indicate that NP elicits depletion of the antioxidant defense system and induces oxidative stress in the brain of mice, which is in accord with the results of Aydogan M. in rats [18]. Evidence suggests that behavioral impairment is a consequence of oxidative stress [27,28]. Excessive accumulation of oxidative products in the brain potentiates neurodegeneration and impairs cognitive function [27]. In our study, NP induced oxidative stress may be implicated as a prime candidate mediating the behavioral impairment and memory deficits. Oxidative stress is also believed to be the central element in the regulation of the apoptotic pathways triggered by environmental stressors (cytotoxic agents, pollutants or toxicants) [29,30]. We reported previously that NP chronic application of NP could sensitize mice brain to apoptosis via suppression of bcl-2 transcription and up-regulation of active caspase-3 [7]. Thus, we presume that NP induced oxidative stress contributes to apoptosis in the brain, which may ultimately be responsible for the disruption of cognitive functions in mice.

## 3. Experimental Section

### 3.1. Animals and Treatments

Young male mice (Kunming strain, white, 3-week-old and with weight 27.23 ± 2.96 g) were purchased from the Branch of National Breeder Center of Rodents (Shanghai). Prior to experiments, mice had free access to food and water and were kept under constant conditions of temperature (23 ± 1 °C) and humidity (60%). Nine mice were housed per cage (47.5 cm × 35 cm × 20 cm) on a 12 h light/dark cycle. After acclimatization to the laboratory conditions for one week, the treated groups were given nonylphenol (a mixture of branched side chains containing 85% p-isomers, Fluka, Buchs, Switzerland) (dissolved in corn oil) daily by oral gavages at 50, 100, 200 mg kg^−1^ d^−1^ for 90 days. Mice in the control group were given the vehicle (corn oil) alone. All experiments were carried out in accordance with China’s legislation on the use and care of laboratory animals and were approved by the respective university committees for animal experiments. After the behavioral testing, mice were sacrificed and brain tissues were immediately collected for experiments or stored at −70 °C for later use.

### 3.2. Behavioral Tests

#### 3.2.1. Open Field Test

The open field test was performed as described by Lu *et al.* [31]. The open field apparatus was a circular arena (50 cm in diameter, 30 cm in height) illuminated with a single 40 W bulb (3000 lux) placed 2.8 m above the bottom center. The bottom of the arena was divided into 21 equal-area grid cells by white lines. Tests were performed in the breeding room from 8:30 to 16:00 h. The individual mice were placed in the middle of the chamber for each trial. After 1-min adaptation, the behavior of each mouse was recorded for 5 min by two observers 1 m away from the open-field area. Between trials, the mice were returned to their home cage in the same room and the open field was wiped clean with a slightly damp cloth. The behavioral parameters recorded were: (1) ambulation: the number of grids crossed in the arena during the observation period; (2) rearing: the number of times the mouse stood on its hind legs; (3) leaning: the number of times the mouse placed one or two forelimbs on the wall of the arena; (4) grooming: the number of times the mouse “washed” itself by licking, wiping, combing, or scratching any part of the body.

#### 3.2.2. Step-Through Test

The trough-shaped step-through passive avoidance apparatus consisted of an illuminated chamber (11.5 cm × 9.5 cm × 11 cm) attached to a darkened chamber (23.5 cm × 9.5 cm × 11 cm) containing a metal floor that could deliver footshocks. The two compartments were separated by a guillotine door. The illuminated chamber was lit with a 25 W bulb. The following test was performed as described by Lu *et al.* [31]. Before training, the mice were placed in the dimly lit room containing the apparatus for 0.5 h to acclimatize to the new environment. During training, each mouse was placed into the illuminated chamber, facing away from the door to the dark chamber to acclimatize for 1 min. When the mouse turned its body fully away from the dark chamber, the door was raised; when the mouse next turned fully toward the darkened chamber, the timer was started. An initial time measure spanned from the time that the mouse faced the opened darkened chamber to the time that the mouse fully entered, with all four paws, the dark chamber. As soon as the mouse entered the dark chamber the door was slid back into place, triggering a mild footshock (0.3 mA, 50 Hz, 5 s). The mouse was then immediately removed from the chamber and returned to its home cage. The retention test was conducted 24 h later with the mouse again being placed in the illuminated chamber and subjected to the same protocol described above in the absence of footshock. The upper time limit was set at 300 s.

#### 3.2.3. Morris Water Maze Test

The Morris water maze test was performed as described by Lu *et al.* [31]. The experimental apparatus consisted of a circular water tank (100 cm in diameter, 35 cm in height), containing water (23 ± 1 °C) to a depth of 15.5 cm, which was rendered opaque by adding ink. A platform (4.5 cm in diameter, 14.5 cm in height) was submerged 1 cm below the water surface and placed at the midpoint of one quadrant. The pool was located in a test room, which contained various prominent visual cues. Each mouse (about 16-weeks-old) received four training periods per day for 4 consecutive days. Latency to escape from the water maze (finding the submerged escape platform) was calculated for each trial. On Day 5, the probe test was carried out by removing the platform and allowing each mouse to swim freely for 60 s. The times that mice spent swimming in the target quadrant (where the platform was located during hidden platform training), and in the three non-target quadrants (right, left and opposite quadrants), were measured, respectively. For the probe trials, the number of times crossing over the platform site of each mouse was also measured and calculated. All data were recorded with a computerized video system.

### 3.3. Biochemical Studies

#### 3.3.1. Tissue Homogenate

For biochemical studies, animals were deeply anesthetized and sacrificed. Brains were promptly dissected, weighed and perfused with 50 mM (pH 7.4) ice-cold phosphate buffered saline solution (PBS). Brains were homogenized in 1/5 (w/v) PBS containing a protease inhibitor cocktail (Sigma-Aldrich, MO, U.S.) using a glass-Teflon homogenizer. Aliquots (2 mL) of this homogenate were directly centrifuged at 8000 × g for 10 min and the supernatant was used to determine brain malondialdehyde (MDA) levels, catalase (CAT), glutathione peroxidase (GPx), glutathione reductase (GR) activities, and protein contents. The remainder homogenate was sonicated four times for 30 s with 20 s intervals using a VWR Bronson Scientific sonicator, centrifuged at 5000 × g for 10 min at 4 °C, and the supernatant was collected and stored at −70 °C for determination of superoxide dismutases (SOD) enzyme activities. Protein concentration was determined by using the BCA assay kit (Pierce Biotechnology Inc., Rockford, IL U.S.) with bovine serum albumin (BSA) used as standard.

#### 3.3.2. Assay of SOD Activity

Chemicals used in the assay, including xanthine, xanthine oxidase, cytochrome c, BSA and SOD, were purchased from Sigma Chemical Company (St. Louis, MO, U.S.). Total SOD was assayed using the method of Flohe and Otting [32]. The activity was measured by the inhibition of cytochrome c reduction mediated via the superoxide anions that were generated by xanthine-xanthine oxidase and assayed spectrophotometrically at 550 nm. One unit of SOD activity was defined as the amount of SOD required for 50% inhibition of the xanthine and xanthine oxidase system reaction in 1 mL enzyme extraction per milligram of protein.

#### 3.3.3. Analysis of CAT Activity

Catalase activity was assayed by the method of Claiborne *et al.* [33]. The assay mixture contained 2.4 mL of phosphate buffer (50 mM, pH 7.0), 10 μL of 19 mM hydrogen peroxide (H_2_O_2_) and 50 μL homogenate. The decrease in absorbance at 240 nm was measured immediately against a blank containing all the components except the homogenate at 10 s intervals for 3 min on a spectrophotometer. The catalase activity was calculated in terms of nmol H_2_O_2_ consumed min/mg protein.

#### 3.3.4. Assay of GPx Activity

The activity of GPx was assayed by the method of Mohandas *et al.* [34]. The assay measures the enzymatic reduction of H_2_O_2_ by GPx through consumption of reduced GSH that is restored from oxidized glutathione GSSG in a coupled enzymatic reaction by GR. GR reduces GSSG to GSH using NADPH as a reducing agent. Disappearance of NADPH was measured immediately at 340 nm against a blank containing all the components except the enzyme at 10 s intervals for 3 min on a Molecular Devices M2 plate reader (Molecular Devices, Menlo. Park, CA U.S.). One unit of GSH-Px was defined as the amount of enzyme that catalyzed the oxidation of 1.0 mmol of NADPH to NADP^+^ per minute at 25 °C.

#### 3.3.5. Analysis of GR Activity

GR activity was measured by the method of Mizuno and Ohta [35]. The enzymatic activity was assayed photometrically by measuring NADPH consumption. In the presence of GSSG and NADPH, GR reduces GSSG and oxidizes NADPH, resulting in a decrease of absorbance at 340 nm, which was measured in a M2 plate reader. Quantification was based on the molar extinction coefficient of 6.22 mM^−1^ cm^−1^ of NADPH. One unit of GR was defined as the amount of enzyme that reduced 1.0 mmol of GSSG (corresponding to the consumption of 1 mmol of NADPH) per minute at 25 °C.

#### 3.3.6. Measurement of Malondialdehyde (MDA) Levels

Chemicals used in the assay, such as n-butanol, thiobarbutiric acid, sodium lauryl sulfate *etc.*, were purchased from Sigma Chemical Company (St. Louis, MO, U.S.). As a marker of lipid peroxidation, the level of MDA was determined as described by Okhawa *et al.* [36]. The brain homogenate (0.2 mL) was mixed with 0.2 mL of 8.1% sodium lauryl sulfate, 1.5 mL of 20% acetic acid (pH 3.5) and 1.5 mL of 0.8% aqueous solution of thiobarbituric acid. The mixture was made up to 4 mL with distilled water and heated at 95 °C for 60 min. After cooling with tap water, 5 mL of *n*-butanol and pyridine (15:1, v/v) and 1 mL of distilled water was added and centrifuged at 4000 rpm for 10 min. The colored complex was extracted into *n*-butanol, and the absorption at 532 nm was measured and MDA content was expressed as nmol/mg protein.

### 3.4. Statistical Analysis

Results are shown as the mean ± S.E. Group differences in the escape latency and swimming speed in the training task of Morris water maze test were analyzed using repeated measures analysis of variance (ANOVA), the factors being treatment and training day. The other data were analyzed by ANOVA using post-hoc Tukey’s test. *p*-values of less than 0.05 were considered significant.

## 4. Conclusions

In summary, in the present study, NP induced cognitive impairment and caused oxidative stress in the mouse brain. On the basis of the present results and the previous study that has shown that NP administration causes apoptosis in mice brain [7], we postulate that NP induced oxidative stress triggers apoptosis, which may be ultimately responsible for the disruption of cognitive functions in mice.
